# Detecting Low Concentrations of Nitrogen-Based Adulterants in Whey Protein Powder Using Benchtop and Handheld NIR Spectrometers and the Feasibility of Scanning through Plastic Bag

**DOI:** 10.3390/molecules25112522

**Published:** 2020-05-28

**Authors:** John-Lewis Zinia Zaukuu, Balkis Aouadi, Mátyás Lukács, Zsanett Bodor, Flóra Vitális, Biborka Gillay, Zoltan Gillay, László Friedrich, Zoltan Kovacs

**Affiliations:** 1 Department of Physics and Control, Faculty of Food Science, Szent Istvan University, 1118 Budapest, Hungary or zaukuu.john-lewis.zinia@hallgato.uni-szie.hu (J.-L.Z.Z.); Aouadi.Balkis@hallgato.uni-szie.hu (B.A.); Bodor.Zsanett@hallgato.uni-szie.hu (Z.B.); Vitalis.Flora@hallgato.uni-szie.hu (F.V.); Gillay.Biborka@etk.szie.hu (B.G.); Gillay.Zoltan@etk.szie.hu (Z.G.); 2 Department of Quality Management, BioTech USA Ltd., 1033 Budapest, Hungary; lukacs.matyas@biotechusa.com; 3 Department of Refrigeration and Livestock, Faculty of Food Science, Szent Istvan University, 1118 Budapest, Hungary; friedrich.laszlo.ferenc@etk.szie.hu

**Keywords:** protein-supplements, chemometrics, fingerprinting, near-infrared, spectroscopy optical-glass, commercial LDPE plastic bag, benchtop, handheld

## Abstract

Nitrogen-rich adulterants in protein powders present sensitivity challenges to conventional combustion methods of protein determination which can be overcome by near Infrared spectroscopy (NIRS). NIRS is a rapid analytical method with high sensitivity and non-invasive advantages. This study developed robust models using benchtop and handheld spectrometers to predict low concentrations of urea, glycine, taurine, and melamine in whey protein powder (WPP). Effectiveness of scanning samples through optical glass and polyethylene bags was also tested for the handheld NIRS. WPP was adulterated up to six concentration levels from 0.5% to 3% *w*/*w*. The two spectrometers were used to obtain three datasets of 819 diffuse reflectance spectra each that were pretreated before linear discriminant analysis (LDA) and regression (PLSR). Pretreatment was effective and revealed important absorption bands that could be correlated with the chemical properties of the mixtures. Benchtop NIR spectrometer showed the best results in LDA and PLSR but handheld NIR spectrometers showed comparatively good results. There were high prediction accuracies and low errors attesting to the robustness of the developed PLSR models using independent test set validation. Both the plastic bag and optical glass gave good results with accuracies depending on the adulterant of interest and can be used for field applications.

## 1. Introduction

Proteins are important nutritional requirements with a recommended dietary reference intake (DRI) of 0.8 g of protein per kilogram of body weight [1]. This amounts to 56 g per day for adults with no rigorous daily activities. People engaged in extensive exercises, however, consume more protein due to their intense energy requirements as a result of physical activity. The fast pace style of living in the urban areas have led to an increased demand in the semi-processed form of protein that is, often consumed as a dietary supplement. A major example of such food supplement is whey protein powder which, is rich in amino acids and is produced from whey.

Whey is the viscous remains after precipitation and curd elimination in the cheese production process. Like milk, whey may have different origins but mainly from animal sources such as cattle, goat, sheep, and buffalo. The most abundant and well-known in terms of manufacturing quantity and financial value is from cow milk [2].

Quality assurance of whey protein supplements have always been a challenge in the food industry but this has been even more worrying after milk related food fraud was ranked second in the food fraud and economic adulteration database [3]. Several analytical methods have been explored for quality assurance of protein-based foods but the most common ones are the combustion methods: Dumas method or Kjeldahl digestion. These rely on detecting residual protein concentrations in food products via their nitrogen (N) content. The methods are dependent on the Kjeldahl conversion factor of 6.25 [4] which, has issues of accuracy and dating back to the nineteenth century. A controversial initiative by the Codex Alimentarius committee published that a standard conversion factor of 6.38 rather than 6.25 should be used to determine protein in milk products [4] but this also translates into extra cost. In 2008, this meant an additional expense of 88.5 million euro affecting the European dairy industry [5]. In addition to technicality and cost, the method is based on the assumption that the average nitrogen content of a protein is about 16% [6] but this is inaccurate because the nitrogen content of proteins certainly depends on their entire amino acid profile. While rich nitrogen-based amino acids such as glycine (18.6% N), histidine (27.1% N), taurine (11.19% N) and melamine (66.6% N), urea (46.62% N) would lower the standard conversion factor, poor nitrogen-based amino acids such as phenylalanine (8.5%) and tyrosine (7.7%) [6] etc., would increase it. Therefore, substituting rich nitrogen-based adulterants into protein powders is a viable adulteration technique. Amino acids such as glycine and taurine are among those that have been recently targeted for protein supplement adulteration because they are generally cheaper full protein sources compared to other common adulterants. Nonetheless, dangerous chemicals such as melamine and urea have also been targeted for their rich nitrogen contents. The intrinsic properties of some of these amino acids makes their adulteration even more potent and difficult to detect. For instance, glycine (H_2_NCH_2_COOH) is considered as an ideal adulterant for protein powders because it is a naturally sweet compound but at high doses, glycine can cause some negative side effects. Taurine (C_2_H_7_NO_3_S) plays essential roles in many physiological activities but in excess, it can act as an eye and skin irritant and cause mild diarrhea and constipation. Urea (NH_2_CONH_2_) is a waste product in mammals [7] and has no nutritional benefits but its high nitrogen content can influence the Kjeldahl conversion factor to greatly boost the pseudo protein content of protein powder when used as an adulterant. Melamine (C_3_H_6_N_6_) is an organic nitrogenous compound synthesized from urea and used in the production of plastics, dyes, fertilizers, and fabrics. Melamine consumption have been linked to nephrolithiasis, chronic kidney inflammation, and bladder carcinoma [8].

There is therefore a need for affordable alternative approaches with better sensitivities that can detect such adulterations. Near infrared spectroscopy (NIRS), a stellar method with non-invasive benefits, capable of real-time analysis is a viable option that can be explored for such purposes. It operates on the principles of interaction of electromagnetic radiation with matter to give a compositional assessment of its constituents. In the near infrared region (700–2500 nm), protein powders can be characterized by certain absorption bands that make it possible to authenticate their quality based on fingerprinting [9]. Fingerprinting generally refers to the study of unique characteristics of a product to help detect any deviation from its original quality. In NIRS, this is achieved by spectral assessment using chemometrics and multivariate data analysis. Chemometrics is a science of extracting information from chemical systems by data-driven means whereas multivariate data analysis is a mathematical discipline used in chemometrics [9]. The techniques can be used to visualize patterns or develop models to classify and predict parameters of interest in a dataset.

NIRS has a wide scope of application from product development to food authentication [10]. It has been used to detect yellow metanil in tamarind powder [11], husk in coffee [12], corn flour in paprika powder [13] etc. In the dairy industry, it has been used to detect plant proteins in skimmed milk [14], melamine adulteration in milk [15], predict urea in milk [16], and predict diverse adulterants in cereal products [17]. The technique was recently used to quantify and predict urea, L-taurine, L-histidine in whey protein powder [18]. It was used to quantify melamine in infant formula sample from different stores [19] and in protein powders [20,21,22,23,24]. All these studies however, involved adulterant concentrations of 1% up to 5% adulteration, thus the need to explore the possibility of using these methods to detect lower adulterant concentrations.

Handheld NIRS devices have also been widely applied for quality control purposes in the food industry [11,25,26]. They present advantages of portability and are capable of direct field applications with less complexities but, it is also important to consider some of their major gaps such as, the unavailability of standard cuvettes or sample holders especially when dealing with liquid or powdered samples. In NIRS analysis, the intrinsic and extrinsic properties of the sample holder can influence the acquired spectral information. The sample holder in this case is any object that holds or stores the sample during scanning. Different sample holders have been used in literature that range from glass cuvettes [27], plastic cuvettes, Quartz [23,28]. Practically, the samples have to be transferred into these sample holders before scanning. This can be time-consuming and sometimes require extra cost. It is also less suitable for commercial applications. There is therefore the need to explore the possibility of directly scanning samples through commercial zip-lock plastic bags as this is often where most food samples are stored before experiments.

The aim of this study is to develop models to rapidly classify and predict low concentrations of urea, glycine, taurine, and melamine in whey protein powder using a benchtop and handheld spectrometer. Furthermore, the aim was to compare the accuracy of the models achieved by scanning the samples trough optical glass and commercial low-density polyethylene (LDPE) plastic bag using handheld spectrometer.

## 2. Results and Discussion

### 2.1. Raw Spectra Analysis

**Figure 1:**
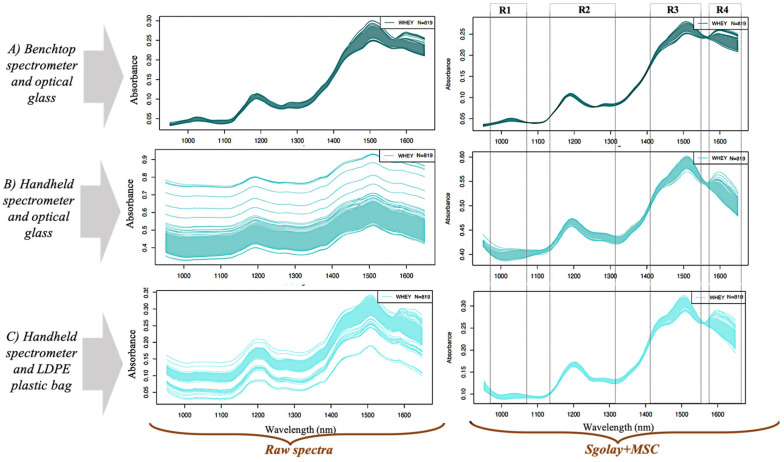
Raw and pretreated diffuse reflectance absorbance spectra plots of pure and adulterated whey protein powder samples scanned with the benchtop spectrometer and handheld spectrometer at the wavelength range of 950–1650 nm.

Figure 1 shows the raw and pretreated spectra of the adulterated protein samples using the benchtop and handheld spectrometer. The handheld spectrometer scanned through the cuvette with optical glass had the highest base line shift variation, its overall base line shift was higher than 0.3 in absorption compared to the one scanned through the commercial LDPE plastic bag and the spectra of the benchtop instrument. The reason for this could be because, the optical glass surface could not fit properly to the window of the handheld spectrometer because of its structural design. This may have resulted in a small air gap between the two surfaces. To reduce detector noise and linear differences among the spectra, Savitzky-Golay smoothing (SGolay) and multiplicative scatter correction (MSC) pretreatments were used and found to be effective in improving the spectra of the handheld spectrometer for both the samples scanned through the optical glass and those scanned through the commercial LDPE plastic bag. The pretreatments did not have a visually clear effect on the spectra of the benchtop instrument as it did with the handheld spectrometer. The wavelength range of 950–1650 nm was found to contain the most important peaks in the spectra from the two spectrometers so this range was chosen for all the other analysis.

In the near infrared region (700–2500 nm), food products and their adulterants can be characterized by certain absorption bands that relay important information about their chemical structure and can be useful for authentication through fingerprinting.

In the absorption spectra in Figure 1, four distinctive regions containing important bands were observed by both the handheld and benchtop spectrometers as shown in Figure 1. The bands observed in region 1 (R1) (950–1160 nm) can be related to the H-O-H third overtone whereas, those in region 2 (R2) (1180–1260 nm) are from the second overtone of C-H stretching [29]. The 1450 nm band in region 3 (R3) is associated with the O-H first overtone from alcohol and is sensitive to H bonding. Also, in R3, the band at 1430 nm corresponds to the second and first overtone regions of N-H bonds or first overtone vibration of water [30]. The band at 1530 nm signifies the presence of either N-H stretching vibration of the amide group from protein or to stretching and O-H [29]. In region 4 (R4), the band at 1570 nm corresponds either to N-H stretching vibrations of amide groups or O-H of vibrating water. The band range 1580–1650 nm corresponds to N-H. Bands 1600–1650 nm signifies the presence of carbonyl groups (C=O).

The chemical structures of the adulterants themselves can be related to some of these important absorption bands in the absorption plot. Melamine for instance, has three nitrogen atoms attached to three amine groups, taurine is characterized by a sulphate group and an amine group, urea is characterized by carbonyl groups (C=O) and two amines, glycine is characterized by a carbonyl group, a hydroxyl group and an amine group. Correlations can be made with the bands in regions 1 to 4 to give a hint about adulterant presence, their mixture combinations or concentrations.

Furthermore, it can be seen from Figure 1 that the benchtop spectrometer gave better spectra with less noise and scattering compared to the handheld spectrometer.

#### 2.1.1. Classification Results of the Benchtop Spectrometer

**Figure 2:**
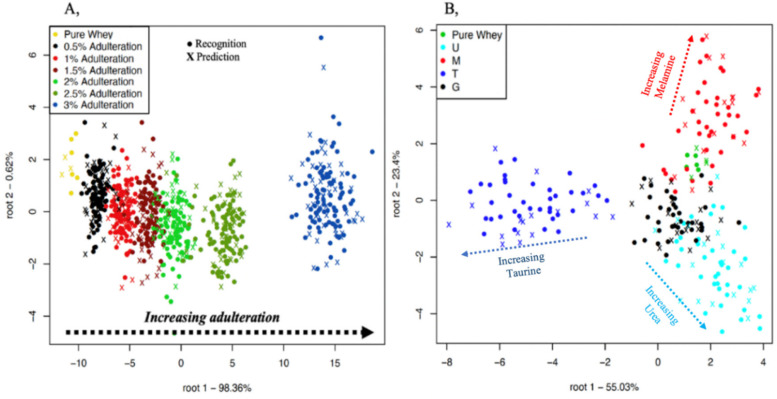
Classification plots of the benchtop spectrometer for adulterant concentration levels (**A**) using the entire dataset and single mixture combinations (**B**) using the data of samples containing single adulterants and pure whey protein powder. U = urea, G = glycine, T = taurine, M = melamine. Spectral pre-processing: Savitzky-Golay smoothing (second order polynomial).

Figure 2A shows the classification plot when the adulterant concentration levels were used as the class variable. There was a clear pattern of separation with increasing adulterant concentration: from the pure whey protein powder to the highest adulterant concentration of 3% *w*/*w* (from left to right in the plot). Good classification results were achieved: average recognition accuracy of 93.64% and prediction of 85.83%. It proves that the pure whey and all the different concentration levels could be successfully distinguished by using the concentration levels as the class variable.

Figure 2B shows the classification plot for the pure and single adulterated mixtures when the adulterant type was used as the class variable. There was a clear pattern of separation between taurine, urine, and melamine. The pure samples were well separated from taurine, and less separated from glycine and urea but overlapped with melamine. There was an average 98.89% recognition and 91.09% prediction accuracy for predicting urea, glycine, melamine, and taurine in single mixture combinations in the whole dataset. It proves that the pure whey could be successfully distinguished from taurine, glycine and urea but not melamine by using the single adulterant types as the class variable.

**Figure 3:**
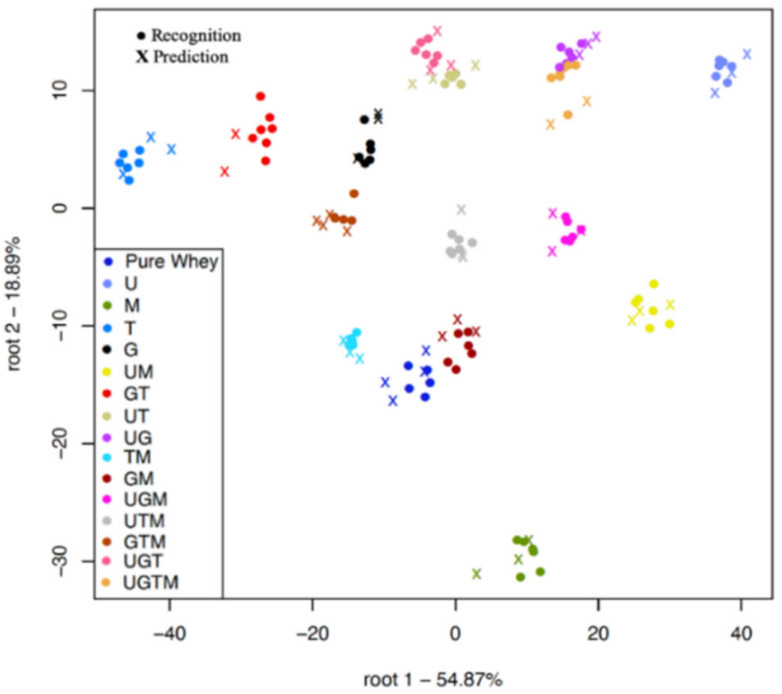
Classification plot of the benchtop spectrometer for the 15-mixture combination using the data of samples with the lowest adulteration level of 0.5% *w*/*w* and the pure whey protein powder. U = urea, G = glycine, T = taurine, M = melamine. Spectral pre-processing: Savitzky-Golay smoothing (second order polynomial).

Figure 3 shows the classification plot of all mixture combinations at the lowest adulterant concentration of 0.5% *w*/*w*. There were 100% average recognition and prediction accuracies that were characterized by a distinct separation of all the mixtures in the classification plot. All the mixture combinations could be clearly separated. The samples containing mixtures with similar components presented inter-group distances smaller than the samples with differing composition, this is particularly clear in the case of samples (UGTM and UG) as well as (UGT and GT).

#### 2.1.2. Classification Results of the Handheld Spectrometer

**Figure 4:**
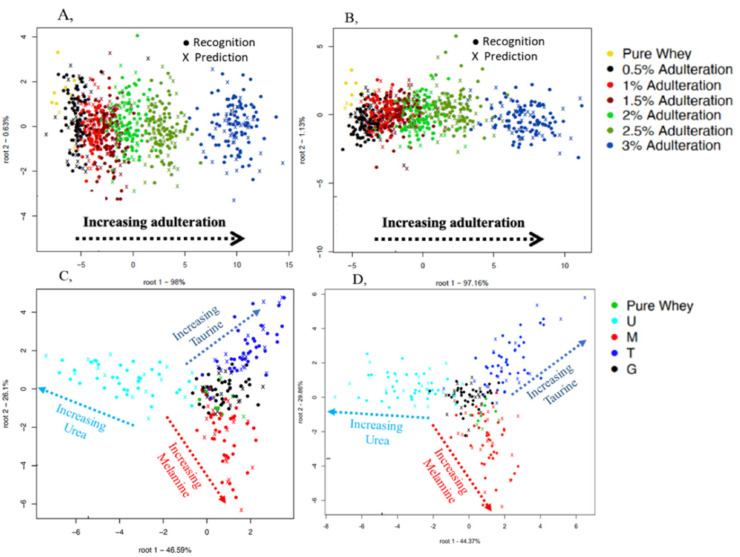
Classification plots of the handheld spectrometer for adulterant concentration levels ((**A**), optical glass, and (**B**) commercial low density polyethylene (LDPE) plastic bag) using the entire dataset and single mixture combinations ((**C**), optical glass, and (**D**), commercial LDPE plastic bag) using the data of samples containing single adulterants and pure whey protein powder. U = urea, G = glycine, T = taurine, M = melamine. Spectral pre-processing: Savitzky-Golay smoothing and multiplicative scatter correction (MSC).

The same separation logic of class variables used for analyzing the spectra from the benchtop spectrometer was also applied. Figure 4 shows four different classification plots using the handheld instrument. Plot A shows the classification according to the different adulterant concentrations when all the adulterated mixtures where scanned through the optical glass whereas, plot B shows the classification when scanned through the commercial LDPE plastic bag. There was an average 76.04% recognition and 58.54% prediction accuracy for detecting concentrations 0.5, 1, 1.5, 2, 2.5, and 3% *w*/*w* urea, glycine, melamine, and taurine in protein powder when scanned through optical glass but higher average accuracies of 80.18% (recognition) and 67.83% (prediction) were achieved when the commercial LDPE plastic bag was used. All the different concentrations could be discriminated in both plot A and B with an increasing pattern of adulterant concentration (Figure 4A,B).

Plot C shows the classification of single adulterants and pure whey protein powder when all the adulterated mixtures were scanned through the optical glass whereas, plot D shows the classification when it was scanned through the commercial LDPE plastic bag. There was an average 97.78% recognition and 85.54% prediction accuracy when scanned through optical glass but lower average accuracies of 93.87% (recognition) and 74.46% (prediction) were achieved when the commercial LDPE plastic bag was used. Although the single adulterants could be discriminated in the classification plots (Figure 4C,D), there was an increasing pattern of concentration from the center of the plot to the extremities that was characterized by some misclassifications between glycine, taurine, melamine, and pure whey protein powder at the lower concentrations.

**Figure 5:**
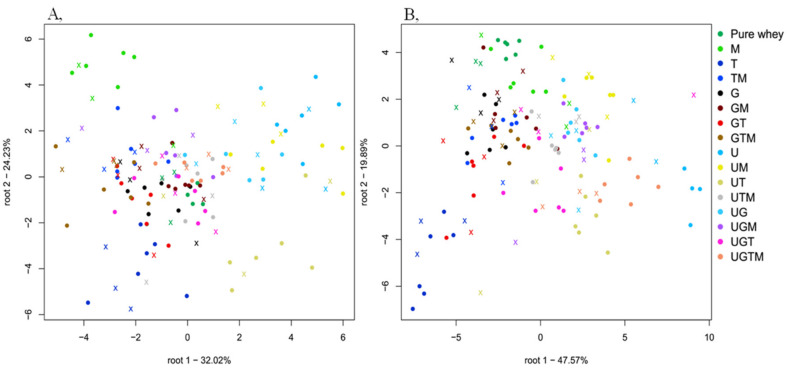
Classification plots of handheld spectrometer for the 15-mixture combination ((**A**) optical glass, and (**B**) commercial LDPE plastic bag) using the data of samples with the lowest adulteration level of 0.5% *w*/*w* and the pure whey protein powder. U = urea, G = glycine, T = taurine, M = melamine. Spectral pre-processing: Savitzky-Golay smoothing and multiplicative scatter correction (MSC).

From Figure 4A,B, the handheld spectrometer could classify all the adulterant concentrations when the whole data set was used, so it was prudent to test the robustness of this classification using only samples having the lowest concentration. This is shown in Figure 5A,B. Average recognition and prediction accuracies of 98.62% and 54.97% respectively were obtained when the optical glass was used. A better average recognition of 99.26% was achieved when the commercial LDPE plastic was used but with a lower average prediction accuracy of 50.44% according to the classification of all mixture combinations in the whole dataset at the lowest adulterant combination of 0.5% *w*/*w*. Both the optical glass and commercial LDPE plastic provided good classification accuracies. There was no clear separation between the mixture combinations but in some cases the single adulterant combinations could be discriminated in the plot. This confirms the complexities and challenge of double and multiple mixture combinations for the handheld instrument at the lowest concentration as observed in Figure 4A,B

### 2.2. Regression Results for Benchtop Spectrometer

Table 1 shows four different PLSR models developed to predict whey protein powder adulteration using the benchtop spectrometer, eight latent variables, and ten-fold cross-validation. 

For the first model (using whole dataset), all the adulterants could be predicted with coefficient of determination after cross-validation (R^2^CV) higher than 0.90 and root means square errors of cross validation (RMSECV) less than 1 g/100 g of whey protein powder. Urea had the highest accuracy after cross-validation with an R^2^CV of 0.95 and a very low error of 0.20 g/100 g.

The prediction accuracies and errors for the second model (using only single adulterant combinations) were similar to those observed in the first model but taurine showed the highest accuracy with R^2^CV of 0.95 and a very low RMSECV of 1.19 g/100 g.

In the third model (using only dual adulterant combinations), the lowest after cross-validation R^2^CV was 0.87 for melamine and with a very low RMSECV of 0.17 g/100 g of whey protein powder. Urea had the highest accuracy with R^2^CV of 0.94 after ten-fold cross validation and a very low RMSECV of 0.19 g/100 g.

For the fourth model (multiple mixture combinations), all the adulterants could be predicted with R^2^CV higher than 0.90 and RMSECV less than 0.61 g/100 g. Urea had the highest accuracy with R^2^CV of 0.92 after 10-fold cross validation and a very low error of 0.13 g/100 g. When the dataset that was not included in the model optimization was used to test the model, a high prediction accuracy (R^2^Pred) between 0.85 and 0.92 was achieved in all the models with highest error (RMSEP) of 1.14 g/100 g. The model containing multiple mixtures showed the best accuracies in both cross-validation and model testing. Taurine had the highest prediction accuracy of 0.95 when only samples with single mixtures were used.

#### 2.2.1. Comparison of PLSR Model Accuracy for Scanning through Optical Glass and Commercial LDPE Plastic Bag with the Handheld Spectrometer

Table 2 shows four different PLSR models developed to predict whey protein powder adulteration by scanning through the commercial LDPE plastic bag, whereas Table 3 shows the models developed with the optical glass.

In cross-validation, the samples scanned through the commercial LDPE plastic bags generally, had better accuracies (highest R^2^CV of 0.93) compared to the R^2^CV 0.91 achieved for those scanned through the optical glass. Samples scanned through the optical glass however, showed the lowest error with RMSECV of 2.08 g/100 g compared to those scanned through the commercial LDPE plastic bag after cross-validation (RMSECV of 2.11 g/100 g). This was also true for the errors when the model was tested with a dataset that was not included in the model calibration. Samples scanned through the commercial LDPE plastic showed the highest prediction errors (RMSEP) of 2.48 g/100 g compared to the 1.84 g/100 g achieved with the optical glass. The highest prediction accuracy (R^2^Pred = 0.91) was achieved for urea in the model with single adulterants. This implies that, although scanning through the commercial LDPE plastic may present better accuracies, it may also present higher errors compared to the optical glass. In cross-validation, urea had the highest R^2^CV’s greater than 0.91 and RMSECV’s lower than 0.34 g/100 g in the model containing multiple adulterants and scanned through the commercial LDPE plastic bag. For those scanned through the optical glass, urea also had the highest accuracy (R^2^CV = 0.91) but this was found in the model containing only single adulterants. The lowest model of 0.17 g/100 g was however, achieved for urea in the model containing multiple adulterants.

For the samples scanned through the optical glass, glycine had the weakest R2CV between 0.58–0.75 for all the models but taurine had the highest RMSECV’s between 1.15–2.08 g/100 g. For the samples scanned through the commercial LDPE plastic bags, glycine also had the weakest R^2^CV’s in the range 0.77–0.76 in all the models except in the first model where taurine was the weakest (R^2^CV = 0.75). Taurine had the lowest error (RMSECV’s) in all the models.

#### 2.2.2. Comparison of PLSR Models Developed for Individual Adulterant based on the Benchtop and Handheld Spectrometers at the Lowest Total Adulterant Concentration of 0.5% *w*/*w*

Benchtop instruments have been generally lauded to have better accuracies than their handheld counterparts, therefore, to ensure that a particular handheld instrument of interest can provide the required level of performance, it is important to correlate or compare its performance to that of the comparable benchtop instrument. Table 1, Table 2 and Table 3 have shown capability of the handheld spectrometer in providing high prediction accuracies comparable to its benchtop counterpart when all the adulteration levels were used. It is no secret that lower concentrations of analytes require higher sensitivity in their detection so this section focuses on the comparison of the benchtop and handheld spectrometers in this regard.

Table 4 shows the different PLSR models developed with the benchtop and handheld spectrometers to predict whey protein powder at the lowest total adulterant concentration of 0.5% *w*/*w*.

The benchtop spectrometer had the strongest model parameters (R^2^CV, RMSECV, R^2^Pred, RMSEP) for predicting all the adulterants but urea had the highest accuracy in cross-validation irrespective of the instrument that was used or the scanning medium. The handheld spectrometer when used with the commercial LDPE plastic bag, could predict urea with the same accuracy (R^2^CV = 0.95) as the benchtop spectrometer at the lowest adulterant concentration of 0.5% *w*/*w*. The handheld spectrometers could also predict all the adulterants with an accuracy (R^2^CV) greater than 0.84 and errors (RMSECV’s) less than 2.11 g/100 g. Good comparison accuracies were also reported by [31] when they also compared the accuracy of a benchtop and handheld diffuse spectrometer in soil measurements and also by [26] for cocaine classification and by [23] in the evaluation of fruit dry matter.

When the dataset that was not used in model optimization was used to test the models, the prediction accuracy diminished in the following order: Benchtop with optical glass, handheld with optical glass then, handheld with commercial LDPE plastic bag. Poor prediction accuracies were achieved for the handheld spectrometer in comparison to the benchtop but the handheld spectrometer equally had low errors of prediction (RMSEP).

## 3. Materials and Methods

### 3.1. Sample Acquisition

Whey protein powder, taurine, and glycine were provided by SCITEC Ltd. (Dunakeszi, Hungary). Urea and melamine were acquired from Elemental SRL (Bihor, Romania) and Sigma-Aldrich Corporation (Darmstadt, Germany), respectively. Whey protein powder was artificially adulterated using melamine (M), urea (U), glycine (G), and taurine (T) as adulterants. The nitrogen content (N) of the adulterants were: melamine (66.60% N), urea (46.62% N), glycine (18.65% N), and taurine (11.19% N). All the powdered samples were prepared to have the same particle size.

### 3.2. Sample Preparation

A combination pattern was developed to contain single adulterant mixtures (U, G, T, M), dual mixtures (GT, GU, GM, TU, TM, UM), and multiple mixtures (GTU, GTM, GUM, TUM, GTUM). This resulted in a total 15 different mixture combinations. All the mixture combinations were prepared to have the total adulteration level (% *w*/*w*) 0.5, 1, 1.5, 2, 2.5, and 3 in whey protein powder. The exact amount of protein powder used in each mixture was calculated based on the nitrogen content of the individual adulterants using melamine as the base adulterant because it had the highest nitrogen content of 66.6% N. Triplicates of each mixture were prepared with each weighing 3 g (total mass after adulteration) and rigorously homogenized, resulting in 273 samples in total. Barcode system was used for easy labelling and identification during scanning with the instruments.

### 3.3. NIRS Measurements

**Figure 6:**
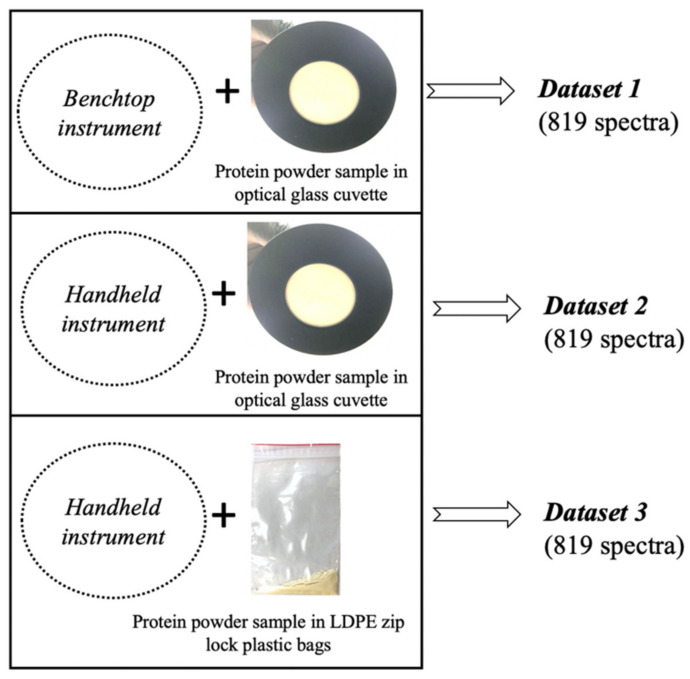
Methods for spectral acquisition of adulterated whey protein powder using the bench top and handheld instrument through a glass window and plastic surface.

The MetriNIR (MetriNIR Research, Development and Service Co., Budapest, Hungary) with a wavelength range of 750–1700 nm and a spectral resolution of 2 nm was used as the benchtop spectrometer. The NIR-S-G1 (InnoSpectra Co., Hsinchu, Taiwan) with a wavelength range of 900–1700 nm and a spectral resolution of 3 nm was used as the handheld spectrometer. Both instruments were used to collect the diffuse reflectance spectra of all 273 whey protein powder samples through an optical glass window cuvette (Figure 6) providing 0.4 mm layer thickness of the tested powders. Three consecutive scans were recorded for each of the three repeats using the two instruments resulting in a total of 819 spectra per instrument, as shown in Figure 6 (dataset 1 and dataset 2). For uniformity of the powdered samples in the glass window cuvette, the cuvette was gently tapped three times against a laboratory work bench before spectral acquisition. Spectral measurements were also recorded for 3 g of each repeat after they were transferred into low density polyethylene (LDPE) zip-lock bags scanned using only the handheld instruments, to give another 819 spectra as shown in Figure 6 (dataset 3). All spectral acquisition was performed at room temperature and the temperature and humidity of the room was monitored using the Voltcraft DL-121TH Multi-Data logger to reveal any substantial environmental condition.

### 3.4. Multivariate Data Analysis of NIRS Spectra

From raw spectra analysis, it was observed that the noisy region corresponded to the lower and higher NIR ranges and that the most important peaks could be seen between the wavelength ranges of 950–1650 nm, so all subsequent analyses were performed within this range. Savitzky-Golay smoothing filter was applied to reduce spectral noise. Multiplicative scatter correction (MSC) was also applied to reduce any possible baseline variations before linear discriminant analysis. Outlier detection was equally tested and no outliers were identified.

#### 3.4.1. Linear Discriminant Analysis

Linear discriminant analysis (LDA) was used for multi-class classification of the different adulterated samples. LDA is a supervised method therefore, the class variable (dependent variable) must be specified in building models. Three different LDA models were developed for each of the three datasets in Figure 6, using three different class variables: (1) The adulterant concentration using the entire dataset, (2) mixture combinations using the data of samples containing single adulterants (U, G, T, M) and pure whey protein powder, (3) mixture combinations using the data of samples that had the lowest adulteration level of 0.5% *w*/*w*.

The predictive significance of each LDA model was tested by splitting the data into two groups: the training set and validation set. The training set consisted of two-third of the data which included spectra from the first and second replicates. The validation set consisted of spectra from the third replicate. The data splitting was repeated three times by substituting the replicates in both the calibration and validation sets. The statistical parameters used to evaluate the performance of the LDA models were the average recognition accuracy for the calibration and average prediction accuracy for the cross-validation.

#### 3.4.2. Partial Least Square Regression

Partial least square regression (PLSR) models were built to test the predictive significance of urea (U), glycine (G), taurine (T), and melamine (M) in whey protein powder using the benchtop and the handheld instruments in five different arrangements. The first model was to ascertain the prediction significance of urea, glycine, taurine, and melamine in whey protein powder adulteration irrespective of the adulterant combination or concentrations, i.e., the whole dataset was used for the modeling. The second model tested the predictive significance of urea, glycine, taurine, and melamine in whey protein powders containing only single adulterants (U, G, T, and M) irrespective of their concentrations. The third model tested the predictive significance of urea, glycine, taurine, and melamine in whey protein powders containing only dual adulterants (UG, UM, UT, GT, TM, and GM) irrespective of their concentrations. The fourth model tested the predictive significance of urea, glycine, taurine, and melamine in whey protein powders containing only triple and quadruple adulterants (UGT, UGM, UTM, GTM, TM, and UGTM) irrespective of their concentrations. Lastly, models were developed to compare the regression accuracies of the handheld and benchtop instrument in predicting urea, glycine, taurine, and melamine at their lowest concentrations of 0.5% w/w irrespective of the mixture combinations.

The statistical parameters used to evaluate the performance of the PLSR models were the root mean square error of calibration (RMSEC) and the coefficient of determination (R^2^C); in ten-fold cross-validation (RMSECV, R^2^CV). The optimum number of latent variables was determined based on the minimum RMSECV value and was kept at 8 to minimize the probability of over fitting.

The robustness of the developed models was finally tested by performing an independent prediction using the same data splitting logic like in LDA where, the test set contains data that was not used during the model optimization process. The accuracies for this test were reported as determination coefficient of prediction (R^2^Pred) and root mean square error of prediction (RMSEP). RMSEC, RMSECV, and RMSEP were expressed as g/100 g equivalent of melamine’s nitrogen content in the protein powder mixtures. The “aquap2” package [32] in R-project was used for all spectral evaluations.

## 4. Conclusions

The pretreated spectra from the benchtop and handheld device revealed important absorption bands can be correlated with the chemical properties of the mixtures.

Classification models were built to predict adulterant concentrations and mixture combinations. Benchtop spectrometer provided the best accuracies in LDA. There were also good average recognition and prediction accuracies when handheld spectrometer was used to develop models for samples that were scanned through both optical glass and commercial LDPE plastic bags. Handheld spectrometers however, had some challenges with classification at 0.5% *w*/*w* adulteration level.

Benchtop spectrometer had the best models in PLSR resulting RMSEP of 0.23, 0.82, 1.14, and 0.21 g/100 g for urea, glycine, taurine, and melamine when all the different combinations of adulteration were used to build the models. Models built based on the spectra acquired by the handheld spectrometer also provided comparatively good performance when either optical glass or plastic bag was used.

The results show the feasibility of rapid classification and prediction or urea, glycine, taurine, and melamine in whey protein powder with high accuracies and low errors using both benchtop and hand spectrometer. Comparatively benchtop spectrometer proved to be the best in predicting these adulterants but handheld spectrometer also provided good and reliable accuracies in LDA and PLSR that can be used to monitor whey protein powder quality. Both optical glass and commercial LDPE plastic bag medium of scanning proved useful for the rapid determination of nitrogen-rich adulterants in whey protein powder.

## Figures and Tables

**Table 1:** Partial least square regression (PLSR) models for adulterated whey protein powder samples scanned with the benchtop spectrometer and analyzed at the spectral range of 950–1650 nm.

Models	Adulterant	R^2^	RMSEC (g/100 g)	R^2^CV	RMSECV (g/100 g)	R^2^Pred	RMSEP (g/100 g)
(1) Whole data	Urea	0.94	0.21	0.94	0.21	0.92	0.23
	Glycine	0.91	0.64	0.90	0.65	0.85	0.82
	Taurine	0.92	0.97	0.92	0.99	0.90	1.14
	Melamine	0.90	0.18	0.90	0.19	0.86	0.21
(2) Model Single mixtures	Urea	0.95	0.28	0.94	0.30	0.92	0.36
	Glycine	0.92	0.90	0.91	0.93	0.82	1.33
	Taurine	0.96	1.08	0.95	1.19	0.95	1.18
	Melamine	0.95	0.20	0.94	0.21	0.91	0.27
(3) Dual mixtures	Urea	0.94	0.18	0.94	0.19	0.94	0.19
	Glycine	0.92	0.53	0.91	0.57	0.91	0.56
	Taurine	0.93	0.86	0.92	0.90	0.87	1.16
	Melamine	0.89	0.17	0.87	0.17	0.87	0.18
(4) Multiple mixtures	Urea	0.93	0.13	0.92	0.13	0.92	0.14
	Glycine	0.92	0.33	0.91	0.34	0.92	0.33
	Taurine	0.91	0.58	0.90	0.61	0.92	0.55
	Melamine	0.92	0.09	0.91	0.09	0.92	0.09

**Table 2:** PLSR models for adulterated whey protein powder samples scanned with the handheld spectrometer through the commercial LDPE plastic bag and analyzed at the spectral range of 950–1650 nm.

Model Dataset	Predicted Adulterant	R^2^	RMSEC (g/100 g)	R^2^CV	RMSECV (g/100 g)	R^2^Pred	RMSEP (g/100 g)
(1) Whole data	Urea	0.93	0.22	0.92	0.23	0.91	0.25
	Glycine	0.79	0.96	0.77	1.03	0.73	1.11
	Taurine	0.78	1.65	0.75	1.77	0.72	1.84
	Melamine	0.79	0.27	0.78	0.28	0.75	0.29
(2) Only Single mixtures	Urea	0.95	0.27	0.93	0.33	0.88	0.42
	Glycine	0.88	1.07	0.77	1.51	0.39	2.48
	Taurine	0.89	1.69	0.83	2.11	0.83	2.08
	Melamine	0.90	0.27	0.86	0.33	0.65	0.51
(3) Only Dual mixtures	Urea	0.95	0.27	0.92	0.33	0.85	0.29
	Glycine	0.88	1.07	0.77	1.51	0.84	0.78
	Taurine	0.89	1.69	0.83	2.11	0.75	1.58
	Melamine	0.90	0.27	0.86	0.33	0.56	0.35
(4) Only Multiple mixtures	Urea	0.95	0.27	0.93	0.33	0.87	0.17
	Glycine	0.88	1.07	0.76	1.50	0.65	0.71
	Taurine	0.89	1.69	0.83	2.11	0.66	1.11
	Melamine	0.90	0.27	0.86	0.33	0.66	1.19

**Table 3:** PLSR models for adulterated whey protein powder samples scanned with the handheld spectrometer through the optical glass and analyzed at the spectral range of 950–1650 nm.

Model Dataset	Predicted Adulterant	R^2^	RMSEC (g/100 g)	R^2^CV	RMSECV (g/100 g)	R^2^Pred	RMSEP (g/100 g)
(1) Whole data	Urea	0.89	0.26	0.89	0.27	0.91	0.25
	Glycine	0.77	0.98	0.75	1.01	0.75	1.03
	Taurine	0.84	1.39	0.82	1.47	0.85	1.37
	Melamine	0.85	0.23	0.83	0.24	0.87	0.21
(2) Model Single mixtures	Urea	0.93	0.32	0.89	0.43	0.82	0.53
	Glycine	0.84	1.22	0.75	1.56	0.80	1.39
	Taurine	0.87	1.86	0.84	2.08	0.87	1.84
	Melamine	0.89	0.29	0.84	0.34	0.73	0.44
(3) Dual mixtures	Urea	0.92	0.22	0.89	0.26	0.79	0.35
	Glycine	0.77	0.81	0.71	0.92	0.77	0.81
	Taurine	0.86	1.18	0.83	1.29	0.84	1.27
	Melamine	0.85	0.20	0.83	0.22	0.77	0.26
(4) Multiple mixtures	Urea	0.88	0.16	0.85	0.18	0.89	0.15
	Glycine	0.72	0.62	0.58	0.76	0.65	0.69
	Taurine	0.76	0.97	0.66	1.15	0.59	1.24
	Melamine	0.77	0.16	0.68	0.19	0.71	0.17

**Table 4:** PLSR models developed with the benchtop and handheld spectrometers for whey protein powder samples containing the lowest concentration of adulterants (0.5%) using a spectral range of 950–1650 nm.

Instrument	Predicted Adulterant	R^2^	RMSEC (g/100 g)	R^2^CV	RMSECV (g/100 g)	R^2^Pred	RMSEP (g/100 g)
Metri benchtop	Urea	0.95	0.28	0.94	0.30	0.96	0.04
	Glycine	0.92	0.90	0.91	0.93	0.84	0.20
	Taurine	0.96	1.08	0.95	1.19	0.89	0.27
	Melamine	0.95	0.20	0.94	0.21	0.22	0.14
Nirscan nano with glass surface cuvette	Urea	0.93	0.32	0.89	0.43	0.57	0.13
	Glycine	0.84	1.22	0.75	1.56	0.42	0.37
	Taurine	0.87	1.86	0.84	2.08	0.56	0.55
	Melamine	0.89	0.29	0.84	0.34	0.24	0.12
Nirscan nano with plastic bag	Urea	0.95	0.27	0.93	0.33	0.22	0.13
	Glycine	0.88	1.07	0.77	1.51	0.34	0.42
	Taurine	0.89	1.69	0.83	2.11	0.51	0.60
	Melamine	0.90	0.27	0.86	0.33	−0.04	0.15
